# A simple tool for evaluation of inflammation in psoriasis: Neutrophil-to-lymphocyte and platelet-to-lymphocyte ratio as markers in psoriasis patients and related murine models of psoriasis-like skin disease

**DOI:** 10.1007/s00109-023-02406-4

**Published:** 2023-12-21

**Authors:** Katharina S. Kommoss, Tabea Bieler, Julia Ringen, Annika Lehmann, Silvia Mihalceanu, Lukas Hobohm, Karsten Keller, Anna Brand, Berenice Fischer, Daniela Kramer, Johannes Wild, Ari Waisman, Alexander Enk, Knut Schäkel, Mathias Heikenwälder, Susanne Karbach

**Affiliations:** 1 Department of Dermatology, University Hospital Heidelberg, Heidelberg, Germany; 2 Division of Chronic Inflammation and Cancer, German Cancer Research Center (DKFZ), Heidelberg, Germany; 3 Interdisciplinary Center for Scientific Computing (IWR), Heidelberg University, Heidelberg, Germany; 4 Center for Thrombosis and Hemostasis, University Medical Center Mainz, Mainz, Germany; 5 Center for Cardiology–Cardiology I, University Medical Center Mainz, Mainz, Germany; 6 Department of Sports Medicine, Medical Clinic VII, University Hospital Heidelberg, Heidelberg, Germany; 7 Institute of Molecular Medicine, University Medical Center Mainz, Mainz, Germany; 8 Department of Dermatology, University Medical Center of the Johannes Gutenberg-University of Mainz, Mainz, Germany; 9 German Center for Cardiovascular Research (DZHK)–Partner Site RheinMain (Mainz), Mainz, Germany; 10 The M3 Research Center, Institute for Interdisciplinary Research On Cancer Metabolism and Chronic Inflammation, Medical Faculty, Eberhard Karls University Tübingen, Tübingen, Germany

**Keywords:** Psoriasis, Psoriasis-comorbidities, Interleukin-17A, Neutrophil-to-lymphocyte ratio, Platelet-to-lymphocyte ratio

## Abstract

**Abstract:**

Objective parameters to quantify psoriatic inflammation are needed for interdisciplinary patient care, as well as preclinical experimental models. This study evaluates neutrophil-to-lymphocyte ratio (NLR) and platelet-to-lymphocyte ratio (PLR) in psoriasis patients and five murine models of psoriasis-like skin disease based on topical imiquimod application and overexpression of IL-17A under different promotors. We performed a single-center prospective observational study in a German population, investigating psoriasis patients prior to, 4 weeks, and 16 weeks post begin of systemic anti-inflammatory therapy. Psoriasis area and severity index (PASI), blood count, and C-reactive protein (CRP) levels were attained at each timepoint. Additionally, five murine models of psoriasis-like skin disease involving five distinct experimental procedures differing in time of disease-onset and severity were investigated regarding PLR and NLR. Of 43 recruited psoriasis patients, 34 patients were followed up to 16 weeks. The cohort was 69.77% male, showing a median age of 32.0 years (range 19.0–67.0; IQR 26). The median PASI decreased from 16.35 (8.0–50.0; 10.20) to 1.6 (0–10.3; 2.56) after 16 weeks of systemic therapy. Spearman’s correlation showed statistically significant positive correlation for NLR with PASI (*r*_*s*_ = 0.27, *p* = 0.006), however not for PLR. NLR, but not PLR, was significantly associated with PASI in a multiple linear regression analysis including age, sex, psoriasis arthritis, and smoking. In the murine models of psoriasis-like skin disease, both NLR and PLR were significantly increased in the acute-severe models compared to controls (*p* < 0.001, *p* = 0.005, and *p* = 0.02, respectively), demonstrating gradually less increased values from severe-acute to mild-late-onset psoriatic phenotype. NLR was significantly associated with PASI in psoriatic patients as well as psoriatic phenotype in different murine psoriasis models. Our data warrants investigation of NLR in psoriasis patients and preclinical psoriasis models as an objective biomarker of psoriatic skin inflammation.

****Key messages**:**

- NLR, but not PLR, showed a statistically significant positive correlation with Psoriasis Area and Severity Index (PASI) in our human psoriasis cohort.
- Both NLR and PLR were significantly increased in murine psoriasis models compared to matched controls, with gradually less increased values from severe-acute to mild-late-onset psoriatic phenotype.
- NLR may represent an easily available, cheap, and objective parameter to monitor psoriatic inflammation in both clinical patient routine, as well as preclinical experimental murine models.

**Supplementary Information:**

The online version contains supplementary material available at 10.1007/s00109-023-02406-4.

## Introduction

Psoriasis is a chronic inflammatory disorder mainly driven by a skewed IL-23/Th17 immune dysregulation [1]. On the skin, psoriasis commonly presents with erythrosquamous plaques, but systemic psoriatic inflammation seems to promote comorbidities such as psoriatic arthritis, cardiovascular disease (CVD), and metabolic changes in the form of elevated blood sugars, cholesterol, and triglycerides [1]. This necessitates interdisciplinary treatment of psoriasis patients, including specialists of dermatology as well as internal medicine, to enable prevention and treatment of associated comorbidities.

To quantify dermatological inflammation in psoriasis, the psoriasis area and severity index (PASI), in which the observer quantifies erythema, scaling, thickness, and expansion of the individual plaques, is widely used among dermatologists [2]. As this is based on subjective assessments, there is the risk of inter-observer-variability. Especially in the setting of clinical trials investigating response to novel therapies, this can be a severe confounder. Additionally, evaluating PASI can be challenging for clinicians lacking daily confrontation with psoriasis patients.

Tools to measure inflammation in psoriasis systemically are up to now not standardized. There is a need for simple, easily accessible, and objective tools to measure systemic psoriatic inflammation for the growing interdisciplinary team of physicians treating psoriasis patients (i.e. general practitioners, cardiologists). As cardiovascular comorbidity in fact forms the life-limiting aspect of psoriasis [3, 4], this holds especially true for interdisciplinary dermatological-cardiological patient care. Furthermore, both in human trials as well as translational research investigating underlying pathomechanisms in psoriasis in preclinical models, objective parameters for quantification of psoriatic inflammation are needed.

Recently, there have been increasing reports on the neutrophil-to-lymphocyte ratio (NLR) and platelet-to-lymphocyte ratio (PLR) as novel markers for systemic inflammation. Thought to reflect the balance between acute or chronic inflammation (via the absolute count of neutrophils or platelets) and adaptive immunity (via the lymphocyte count), they have been investigated as a potential prognostic marker in diseases ranging from infectious, oncologic, cardiovascular, to autoimmune diseases — including psoriasis [5–8]. Interestingly, an increased NLR was described to be associated with CVD outcome [9]. Whether NLR or PLR correlates with the *severity* of psoriasis, as well as *longevity* of psoriatic disease in patients, remains unclear. Additionally, to our knowledge, reports on NLR and PLR within murine models of psoriasis are lacking. We therefore prospectively investigated a single-center cohort of psoriasis patients undergoing systemic psoriasis-specific treatment. Furthermore, we examined multiple murine models of psoriasis-like skin disease (based on two underlying pathomechanisms — either imiquimod treatment or IL-17A overexpression) differing in time of disease-onset and severity regarding their PLR and NLR values: (i) the *acute, severe psoriasis-like dermatitis model* by applying the topical toll-like receptor 7/8 agonist imiquimod (IMQ) for 6 consecutive days [10]; (ii) a *prolonged acute, severe psoriasis model* by extending the IMQ application to 10 days [11]; (iii) the K14-IL-17A^ind/+^ mice with keratinocyte-specific IL-17A overexpression leading to an *early-onset, chronic and severe psoriatic phenotype* [12, 13]; and *two models of delayed-onset, chronic psoriatic phenotype *via IL-17A overexpression in CD11c^+^ cells leading to a moderate ((iv) homozygous CD11c-IL-17A^ind/ind^) to mild ((v) heterozygous CD11c-IL-17A^ind/+^ mice) psoriasis-like skin disease [14]. These are — as all mouse models of human disease — artificial [15] but nevertheless offer the opportunity to investigate potential translational value of NLR and PLR for disease severity assessment in preclinical studies.

## Material and methods

### Ethics

This study was conducted according to the Declaration of Helsinki. The study was approved by the ethics committee of Heidelberg (approval no. S-834/2020), and patients provided written and informed consent.

### Human samples

Patients with confirmed psoriasis diagnosis by a dermatologist, eligible for systemic psoriatic therapy (PASI ≧ 10, upgrade criteria, e.g., scalp or nail involvement, or Dermatology Life Quality Index (DLQI) ≧ 10) and naïve to or post a 12-week washout of previous systemic psoriatic therapy, were included. Exclusion criteria were the presence of additional autoimmune, infectious, or malignant diseases; age < 18 years, pregnancy; and use of anti-platelet or anti-coagulant drugs. Overall, 43 subjects were enrolled in our study. Patient information and blood samples were taken prior to, 4 weeks. and 16 weeks post begin of systemic therapy, leading to three study visits overall within the in- and outpatient clinic in the Department for Dermatology, University of Heidelberg, Germany. The responsiveness to biological therapy was assessed by psoriasis area and severity index (PASI). Systemic therapy (ixekizumab, secukinumab, brodalumab, guselkumab, tildrakizumab, risankizumab, adalimumab, ustekinumab, dimethyl fumarate) was chosen by the treating physician based on intraindividual patient characteristics according to European and national guide-line recommendations and administered accordingly [16, 17].

### Mice

Experiments were approved by the Animal Care and Use Committee from Rhineland-Palatine (Landesuntersuchungsamt Rheinland-Pfalz (LUA), Landau, approval no. G17-1–076) and Baden-Wuerttemberg (Regierungspräsidium Karlsruhe, Karlsruhe, approval no. G249/20). Guidelines from Directive 2010/63/EU of the European Parliament on the protection of animals used for scientific purposes were followed. Mice of both sexes, as well as matched controls, were used. In short, the topical toll-like receptor 7/8 agonist imiquimod (IMQ) was applied to the shaved back and ears for 6 or 10 consecutive days before analysis of the peripheral blood [10]. The previously described transgenic psoriasis model K14-IL-17A^ind/+^ [12, 13], CD11c-IL-17A^ind/ind^, and CD11c-IL-17A^ind/+^ [14] were analyzed at the age of 7 to 26 weeks, depending on onset of phenotype [18].

### Clinical biochemistry, blood parameters, and SAP-ELISA

Human blood count was retracted from electronic patient records. Regular CRP (mg/l) is censored below 2 mg/l. For murine studies, blood was measured in an automated hematometer (Element HT5 (Scil animal care company, Viernheim, Germany) and VetScan HM5 (Abaxis, Union City, CA, USA)). NLR and PLR were calculated by neutrophil and platelet count divided by the lymphocyte count, respectively. Serum amyloid P (SAP) was measured using the Mouse Pentraxin 2/SAP Quantikine® ELISA (R&D Systems, Minneapolis, MN, USA), according to the manufacturer’s protocol.

### Statistical analysis

Statistical analysis was performed with GraphPad Prism software (version 9; GraphPad Software Inc., San Diego, CA, USA). Data are displayed as mean ± standard error of the mean (S.E.M). Data were analyzed for normal distribution (D’Agostino & Pearson test). As this test was significant, nonparametric Kruskal–Wallis test with Dunn’s multiple comparison or comparison of selected columns was used. For matched comparisons, Friedman test or mixed effects analysis with Geisser-Greenhouse correction was used, if single values were missing. We used nonparametric Spearman correlations or simple linear regression where appropriate. A multiple linear regression was performed for both NLR and PLR as dependent variables including age, sex, psoriasis arthritis, and smoking for the primary timepoint of inclusion. *p* values of < 0.05 were considered significant and marked by asterisks (*p < 0.05; **p < 0.01; ***p < 0.001).

## Results

### Patient cohort characteristics

Of the 43 patients included in the study, 69.78% were male and showed a median age of 32.0 years (range 19.0–67.0; IQR 26) (see Table 1). Median disease duration at the time of study inclusion was 8.0 years (0.5–48; 17.75). Patients displayed a median body mass index (BMI) of 30.45 (18.37–53.46; 9.31), 25.58% suffered from additional psoriasis arthritis (PsA), 11.63% from arterial hypertension, 4.65% from diabetes mellitus type II, and 55.81% were smokers. Of the 43 patients, 34 patients were followed up prospectively over the time course of up to 16 weeks post systemic therapy start. The median PASI decreased from 16.35 (8.0–50.0; 10.20) to 4.2 (0.0–14.4; 3.38) to 1.6 (0–10.3; 2.58) after 4 and 16 weeks of systemic therapy, respectively. Targets of systemic therapy were IL-17A in 39.53%, IL-23 in 25.58%, TNF-α (adalimumab) in 9.30%, and anti-IL-12/IL-23 (ustekinumab) or dimethyl fumarate (DMF) in 2.33%.

**Table 1:** Overview over patient characteristics (total cohort *n* = 43)

	***n* (%)** *or*** median (range; IQR)**
**Male sex**	30 (69.77)
**Age** [years]	32.0 (19.0–67.0; 26)
**BMI**	30.45 (18.37–53.46; 9.31)
**Disease duration** [years]	8.0 (0.5–48; 17.75)
**Psoriasis arthritis**	11 (25.58)
**Art. hypertension**	5 (11.63)
**Diabetes mellitus type II**	2 (4.65)
**Smoking**	24 (55.81)
**PASI pre-therapy**	16.35 (8.0–50.0; 10.20)
**PASI 4 weeks post therapy**	4.2 (0.0–14.4; 3.38)
**PASI 16 weeks post therapy**	1.6 (0–10.3; 2.58)
**Anti-IL-17 therapy** Ixekizumab Secukinumab Brodalumab	17 (39.53) 14 (32.56) 2 (4.65) 1 (2.33)
**Anti-IL-23 therapy** Guselkumab Tildrakizumab Risankizumab	11 (25.58) 4 (9.30) 4 (9.30) 3 (6.98)
**Anti-TNF-α therapy** Adalimumab	4 (9.30)
**Anti-IL-12/23 therapy** Ustekinumab	1 (2.33)
**Dimethyl fumarate therapy**	1 (2.33)

*BMI* body mass index, *PASI* psoriasis area and severity index, *IQR* interquartile range

### Analysis of systemic inflammation markers in psoriasis patients

**Fig. 1:**
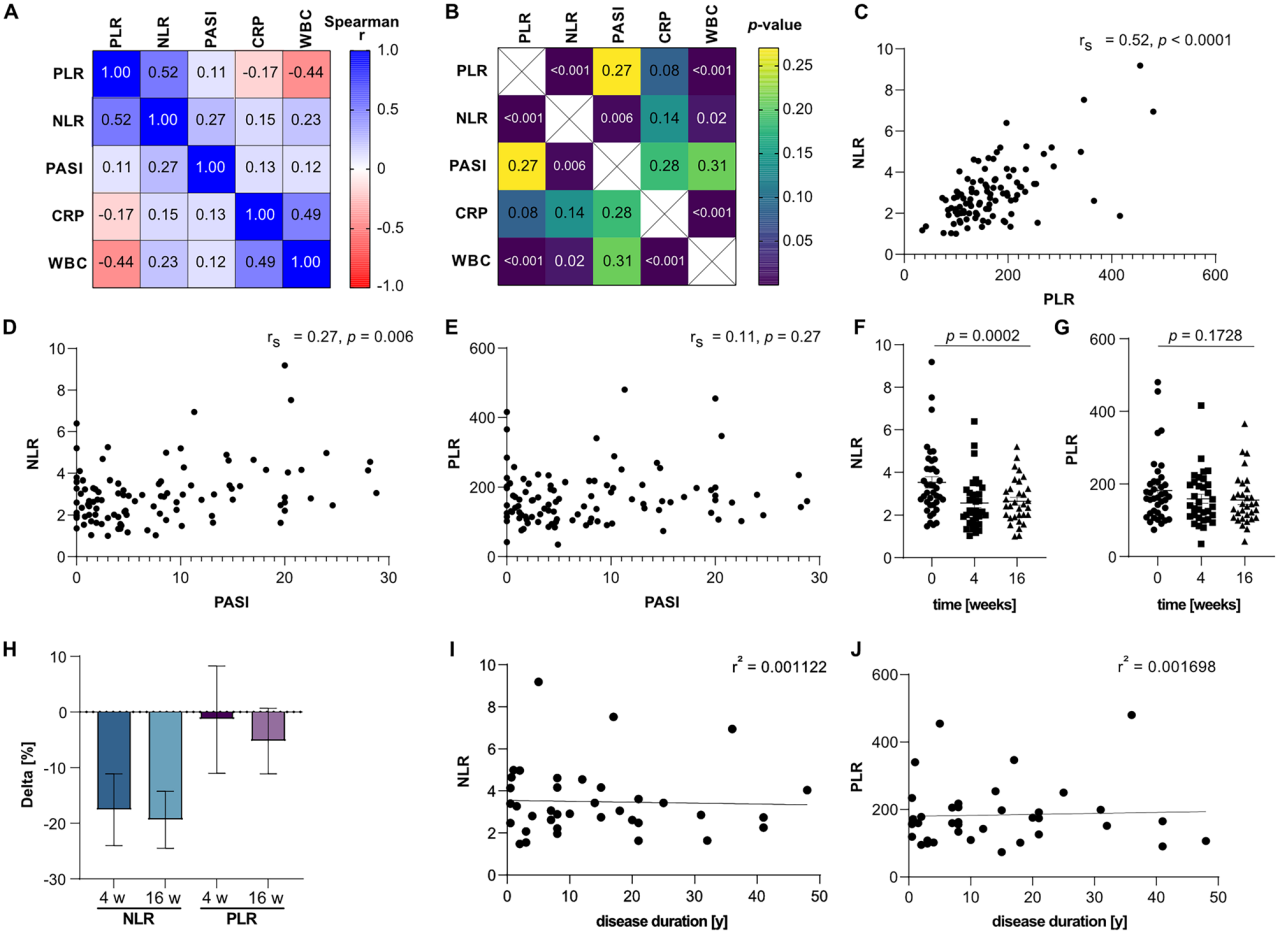
NLR and PLR in human psoriasis cohort. **A** Correlation matrix of PLR, NLR, PASI, CRP, and WBC with respective Spearman’s rho (*r*_*s*_). **B** *p* values of respective correlations plotted in **A**. **C** Correlation of NLR and PLR, *r*_*s*_ = 0.52, *p* < 0.0001. Correlation of NLR (*r*_*s*_ = 0.27, *p* = 0.006) **D** and PLR (*r*_*s*_ = 0.11, *p* = 0.27) **(E)** with PASI. Grouping of NLR **(F)** and PLR **(G)** by time points of study. **H** Delta of NLR and PLR in % compared to baseline for 4 weeks and 16 weeks of therapy, respectively. Simple regression of NLR (*r*^2^ = 0.001122) **(I)** and PLR (*r*^2^ = 0.001698) **(J)** over disease duration. Spearman’s correlation in **A**–**E.** Mixed effect analysis for matched pairs in **F** and **G**. Simple linear regression in **I**–**J**

Spearman’s correlation showed statistically significant positive correlation for NLR and PLR (*r*_*s*_ = 0.52, *p* < 0.0001; Fig. 1A, B, C), as well as for WBC and CRP (*r*_*s*_ = 0.49, *p* < 0.001) in psoriasis patients. Values for PASI correlated with NLR (*r*_*s*_ = 0.27, *p* = 0.006; Fig. 1D), however not with PLR (Fig. 1E), CRP, and WBC (Fig. 1A, B). Both NLR and PLR correlated with WBC, however in a positive (*r*_*s*_ = 0.23) and negative way (*r*_*s*_ = − 0.44), respectively. Multiple linear regression analyses including age, sex, psoriasis arthritis, and smoking for the pre-therapy timepoint showed statistically significant associations with PASI for NLR, but not for PLR (Suppl. Table 1). Analysis of absolute count of neutrophils, platelets, and lymphocytes showed no association with PASI in correlation analysis (Suppl. Figure 1A-C). Grouping of values for the therapeutic time points showed a significant decrease of NLR (Fig. 1F), but not for PLR (Fig. 1G) over time of systemic therapy in a repeated measure matched analysis on an intraindividual level. In addition, this was reflected by the negative intraindividual delta for NLR- and (to a lesser extent) for PLR-values over time of anti-psoriatic therapy (Fig. 1H). There was no association apparent for NLR (Fig. 1I) or PLR (Fig. 1J) with the duration of disease at the baseline timepoint of maximum PASI before starting systemic therapy.

### Analysis of systemic inflammation markers in murine models of psoriasis-like skin disease

**Fig. 2:**
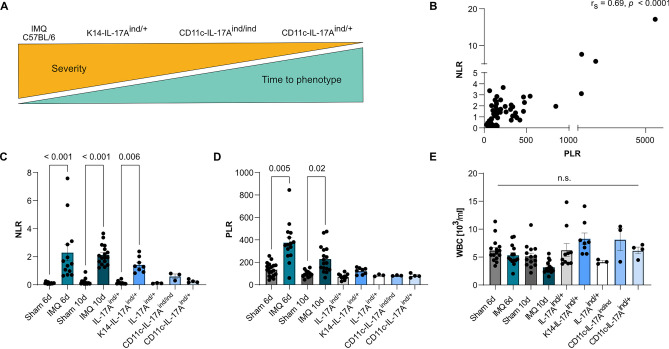
NLR and PLR in different murine psoriasis models. **A** Schematic overview over investigated murine psoriasis models, described previously. **B** Correlation of NLR with PLR in murine psoriasis models (r_s_ = 0.06862, *p* < 0.0001). **C** NLR, **D** PLR, and **E** WBC in different murine psoriatic dermatitis models. Spearman’s correlation in **B**, Kruskal–Wallis test corrected with Dunn’s test for multiple comparisons **(C**–**E)**. *n* = 3–17 per group

We analyzed five murine models of psoriasis-like dermatitis based on topical IMQ application and IL-17A overexpression under different promotors, described previously (Fig. 2A) [10, 11, 13, 14]. To corroborate systemic inflammation, serum amyloid P (SAP) was measured in the respective mouse models (Suppl. Figure 2A). Increased values were seen for all investigated models in comparison to matched control animals. In addition, levels of SAP gradually decreased with severity in the transgenic models based on IL-17A overexpression, as well as with chronicity for the topical imiquimod model. NLR and PLR correlated with each other significantly (Fig. 2B, *r*_*s*_ = 0.69, *p* < 0.0001). Both NLR and PLR were significantly increased in the IMQ-induced psoriasis models compared to sham-treated controls, with higher values in the acute 6-day versus 10-day application model (Fig. 2C, D). Regarding the chronic models of psoriasis-like skin disease, elevated NLR and PLR values were observed in K14-IL-17A^ind/+^, with a decrease of the difference to control mice from K14-IL-17A^ind/+^, to CD11c-IL-17A^ind/ind^, and to CD11c-IL-17A^ind/+^ (Fig. 2C, D). No difference in WBC (Fig. 2E) was seen across models and conditions. Additionally, absolute values for neutrophils, platelets, and lymphocytes did not follow the same trend as observed for NLR and PLR (Suppl. Figure 2B-D).

## Discussion

Increasing interdisciplinary treatment of psoriasis patients due to better understanding of comorbidities and wide-spread use of systemic psoriasis therapies necessitate objective parameters to monitor psoriatic inflammation. Systemic markers for psoriatic inflammation such as inflammatory cytokines (i.e., IL-17A, IL-6, TNF-α) are not part of clinical routine and time- and cost-consuming [19].

We therefore analyzed psoriasis patients before starting established systemic therapy (targeting IL-17A, IL-23, TNF-α, IL-12/23, and DMF) and for a follow-up up to 16 weeks in a single-center prospective study. In parallel, we investigated five murine models of psoriasis-like skin disease, based on topical IMQ application and overexpression of IL-17A under different promotors. While the examined mouse models do reflect hallmarks of psoriasis (each to different extents), as artificial systems with manipulated cytokines/immune cells, they do not recapitulate the human situation congruently. The discussion on how to choose the most valid mouse model and their translational value regarding human disease is continuously ongoing [15]. With the plethora of murine psoriasis models (see Gudjonsson et al. [20] and Gangwar et al. [21]), it is of highest importance to obtain reliable, objective proxies for psoriatic inflammation in preclinical models. Particularly, if collected murine and human data goes hand in hand, this opens avenues for valuable translational insights.

Interestingly, the well-established markers for systemic inflammation CRP and WBC showed no intraindividual correlation with PASI during a 4-month time course of systemic therapy in psoriasis patients (Fig. 1A, B). This is in contrast to the conclusion of a review on the topic of CRP and psoriasis, which inferred CRP to be interchangeable with PASI as a measure of disease severity in patients without psoriatic arthritis [22], and stresses the necessity for further inflammatory markers. However, as we investigated intraindividual changes of inflammatory markers, no comparison to healthy controls or patients with mild psoriasis (PASI < 5) can be drawn. The absent correlation of WBC with PASI seen in the patient cohort was corroborated across all mouse models (Fig. 2E). These findings in patients and murine psoriasis models suggest absolute WBC to be unfit to reflect psoriatic inflammation.

The role of neutrophils, partly driven by the neutrophil-recruiting cytokine IL-17A, is well established as a key driver in psoriatic disease [23]. Their function in the context of neutrophil extracellular traps and reactive oxygen species formation in the psoriatic plaque of the skin has been further investigated in systemic psoriatic inflammation, i.e., cardiovascular inflammation [11, 13, 18]. Additionally, the inflammatory function of platelets has recently been gaining attention in psoriatic research [24, 25], beyond mere reactive thrombocytosis [26]. However, as intraindividual cell counts may vary, the ratio of neutrophils/platelets to lymphocytes may provide an intra-individually normalized value enabling better inter-patient comparison and provide context to acute-vs.-chronic inflammation. A meta-analysis from 2019 by Paliogiannis et al. [6] comes to the conclusion that both NLR and PLR are increased in psoriasis compared to healthy controls. Reports on NLR and PLR as follow-up markers during systemic therapy for psoriasis vulgaris are to date mostly limited to TNF-α inhibitors (etanercept, infliximab, adalimumab [27–29]) or the IL-12/23 inhibitor ustekinumab [27–29], with little data on anti-IL-17A therapy (brodalumab [30], secukinumab, ixekizumab [29]) and IL-23 inhibitors [31]. Therefore, this study extends first data on these values, especially regarding IL-23 inhibitors (guselkumab, tildrakizumab, risankizumab). Interestingly, both values have also been investigated in the context of cardiovascular disease. Elevated NLR demonstrated a predictive value for hypertension incidence [32, 33]. Prognostically, a recent multicenter study found high NLR and PLR values to be independently associated with cardiac death in patients with acute decompensated heart failure [34]. Additionally, a multicenter study found increased baseline NLR to represent an independent predictive biomarker for incident major adverse cardiovascular events in five randomized controlled trials investigating cardiovascular anti-inflammatory drugs (canakinumab, rosuvastatin, bococizumab, or methotrexate) [35].

In patients, NLR, but not PLR, decreased in parallel with PASI under systemic psoriasis treatment in a statistically significant matter (Fig. 1D–H), corroborating previous reports showing increased NLR in psoriasis patients [27–30]. Interestingly, absolute count of neither peripheral lymphocytes, neutrophils, nor platelets correlated with PASI in our human cohort (Suppl. Figure 1B, C), highlighting the potential value of NLR. The findings within the patient cohort were confirmed in the analyzed murine psoriasis-like skin disease models, showing statistically significant elevated ratios in both NLR and PLR (Fig. 2C, D). As reflected in systemic SAP levels (Suppl. Figure 2A), a gradual decrease of NLR and PLR from the severe-acute to mild-late-onset models was apparent. The effect was more pronounced for NLR than PLR, mirroring and underlining the findings in the human cohort. While some studies demonstrated elevated PLR in psoriasis patients [6, 27, 36] and the evidence of a role of platelets in inflammatory diseases, specifically psoriasis, is increasing [24], our findings underscore the conclusion of other reports [37, 38], indicating PLR to not be a marker to well reflect severity of psoriatic inflammation. Further studies are needed to definitively clarify the role of PLR in psoriasis.

Of note, just recently, NLRs (and the cytokine IL-6) were found to be predictive biomarkers in psoriasis patients for treatment response under TNFalpha inhibitors [31]. Thus, this biomarker might serve to individualize and personalize regulation of biologic treatment in autoimmune disease. The next step will be to analyze these markers also in regard to associated cardiovascular disease in psoriasis patients in a long-term approach.

Additionally, to our knowledge, we are the first to investigate NLR and PLR in the context of psoriatic disease duration. With a large range of 0.5 to 50 years of history of psoriasis, we found no correlation for NLR or PLR with disease duration (Fig. 1H, I). In contrast, the murine data may indicate NLR and PLR to be higher in early-onset compared to late-onset disease. However, no clear differentiation between acuteness and disease duration can be drawn due to the experimental setup of the murine models [10, 11, 13, 14]. Taken together, this study suggests both NLR and PLR to not correlate with psoriatic disease duration.

Most likely, NLR and PLR are not disease specific, such as other inflammatory markers as CRP. However, it is noteworthy that only NLR correlated positively with psoriatic dermal inflammation quantified via PASI. Importantly, these easily attained hematological parameters showed correlation with psoriatic disease severity and disease onset in murine models, rendering these values applicable to study psoriatic phenotype and therapeutic responses in preclinical models. Recently, Dey et al. demonstrated a significant correlation of NLR with PASI under anti-TNF-α (adalimumab, etanercept), anti-IL-12/23 (ustekinumab), or anti-IL-17A (secukinumab, ixekizumab) therapy in 316 psoriasis patients [29]. This study underscores the relevance of NLR, as the median PASI was lower in this study than in our study cohort (baseline 6.0 (3.1–11.7) *versus* 16.35 (8.0–50.0), respectively), and the authors further reported an association of NLR with non-calcified coronary artery burden by coronary computed tomography angiography within psoriasis patients. The latter point might hint towards NLR not only reflecting dermal inflammation, but potentially acting as a marker for cardiovascular disease in psoriasis patients. This warrants further investigation into this easily attainable marker in local and systemic psoriatic inflammation.

## Conclusion

In conclusion, we demonstrate NLR — but not PLR — to correlate with PASI intra-individually in psoriasis patients. This correlation was corroborated in five murine models of psoriasis-like skin disease based on topical imiquimod application and IL-17A overexpression under different promotors, in which the elevation of NLR — but not PLR — appeared to reflect severity and early-onset of psoriatic inflammation. Collectively, our findings highlight NLR as an easily accessible, cheap and convenient proxy for psoriatic inflammation in patients and preclinical psoriasis models.

### Limitations

The reported data cannot address a causal understanding for the associations seen for NLR, PLR, and PASI. We have not investigated additional markers for organ-specific inflammation, i.e., cardiovascular inflammation. Additionally, our patient cohort was of small sample size (preventing comparisons of effects of individual therapeutic target classes and leading to underpowering multivariate analyses), predominantly male, and the biologic applied based on the physician’s choice. Therefore, results in the human cohort based on the German population must be seen as hypothesis-generating. Nevertheless, especially NLR might be a good clinical marker for classification of disease severity in psoriasis patients and related murine models of psoriasis-like skin disease. As murine models of human disease have to be considered cautiously, further corroboration of our data in additional murine psoriasis models independent of topical treatment or IL-17A overexpression is needed.

### Supplementary Information

Below is the link to the electronic supplementary material.

Supplementary file1 (JPG 137 KB)

Supplementary file2 (PNG 214 KB)

## Data Availability

The datasets generated during and/or analyzed during the current study are not publicly available due to the ethical permits under which the data was generated, but are available from the corresponding author on reasonable request.

## Abbreviations

BMI: Body mass index
CRP: C-reactive protein
CVD: Cardiovascular disease
DLQI: Dermatology Life Quality Index
DMF: Dimethyl fumarate
IL-12: Interleukin-12
IL-17A: Interleukin-17A
IL-23: Interleukin-23
IMQ: Imiquimod
IQR: Interquartile range
LYM: Lymphocytes
NEU: Neutrophils
NLR: Neutrophil-to-lymphocyte ratio
PASI: Psoriasis area and severity index
PLR: Platelet-to-lymphocyte ratio
PLT: Platelets
PsA: Psoriasis arthritis
SAP: Serum Amyloid P
SD: Standard deviation
S.E.M.: Standard error of the mean
T.N.F-$\alpha$: Tumor necrosis factor alpha
WBC: White blood cells
