# Supplementary figures and images for: A simple tool for evaluation of inflammation in psoriasis: Neutrophil-to-lymphocyte and platelet-to-lymphocyte ratio as markers in psoriasis patients and related murine models of psoriasis-like skin disease
Source: J Mol Med (Berl). 2023 Dec 21;102(2):247–55. doi: 10.1007/s00109-023-02406-4 (PMC10857970; doi:10.1007/s00109-023-02406-4)

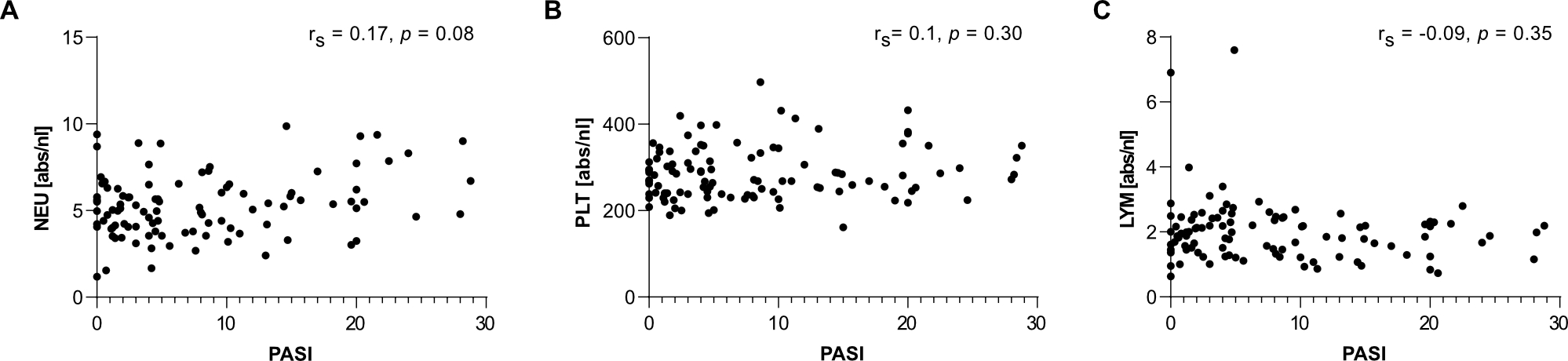

Supplement: Supplementary file 1 — Supplementary file1 (JPG 137 KB) [file 109_2023_2406_MOESM1_ESM.jpg]

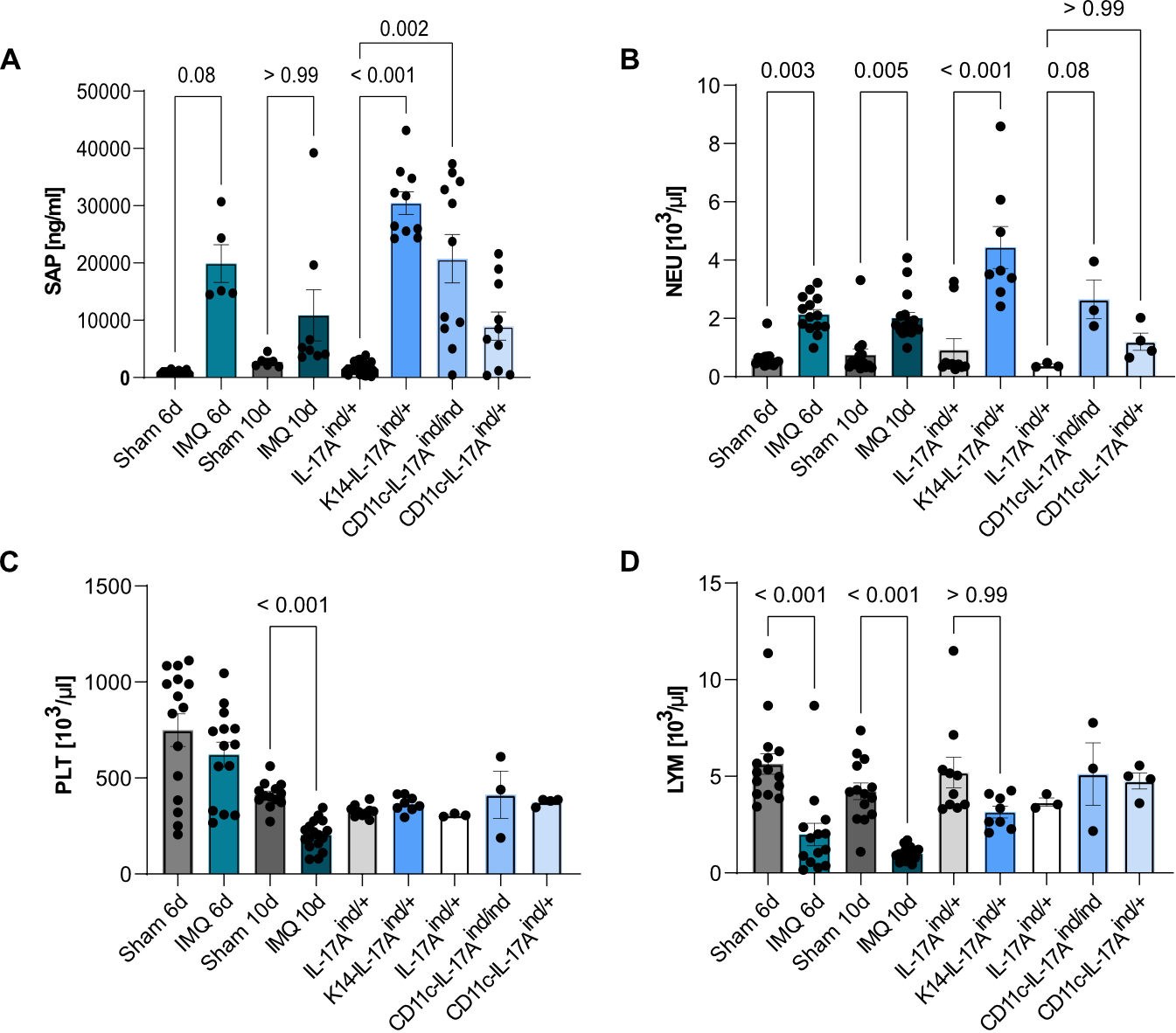

Supplement: Supplementary file 2 — Supplementary file2 (PNG 214 KB) [file 109_2023_2406_MOESM2_ESM.png]
